# Examining Challenges and Evaluating Supportive Counseling Approaches for Students with Attention Deficit Hyperactivity Disorder (ADHD)”

**DOI:** 10.1192/j.eurpsy.2024.1433

**Published:** 2024-08-27

**Authors:** I. Farmakopoulou, A. Metaxa, M. Theodoratou

**Affiliations:** ^1^social work and educational sciences, University of Patras; ^2^Social Sciences, Hellenic Open University, Patras, Greece; ^3^Health Sciences, Neapolis University Pafos, Pafos, Cyprus

## Abstract

**Introduction:**

Introduction: Research indicates that 2-8% of students exhibit ADHD symptoms, a condition impacting personal, social, and academic functionality (Kwon et al., 2018). A significant proportion encounter educational and socio-emotional challenges, often leading to academic disruptions; indeed, 11-21% of such students defer enrollment for two consecutive years (DuPaul et al., 2021). Studies have highlighted pronounced issues related to academic performance within this demographic (Henning et al., 2022). These findings emphasize the critical need for innovative interventions and a deeper understanding of ADHD’s impact on young adults in academic contexts.

**Objectives:**

To investigate challenges and evaluate supportive counseling approaches for students with Attention Deficit Hyperactivity Disorder (ADHD)”

**Methods:**

This study utilized a qualitative approach, employing semi-structured interviews to understand the experiences and perspectives of university students with ADHD from across the country. The diverse sample comprised students from various academic disciplines and levels. Data were collected, ensuring participants’ comfort, and were analyzed using content analysis method, revealing insightful themes and patterns about ADHD’s impact in students’ quality of life and academic issues. The findings aim to contribute to a better understanding of ADHD

**Results:**

The results of the study highlight the significant academic and organisational difficulties faced by participants with ADHD. Many struggled intensely to maintain concentration in class, with distractions causing significant attentional lapses and increased anxiety. Procrastination was a recurring problem, leading to last-minute submissions and increased stress. Forgetting to complete academic tasks, such as course registration, had a cumulative negative impact on participants’ academic journeys. These findings highlight the complex challenges faced by people with ADHD in educational settings, and the need for comprehensive interventions. Addressing these multifaceted issues goes beyond academic accommodations and requires inclusive learning environments, counselling, peer support, and specialised faculty training to create a supportive ecosystem conducive to the success of individuals with ADHD.

**Conclusions:**

This study highlights the multifaceted challenges, notably in concentration and task management, faced by individuals, presumably with ADHD, within academic settings. The reported struggles emphasize the urgent need for specialized interventions and support structures, focused on fostering concentration, effective task management, and administrative diligence. The insights provided are instrumental, guiding future research and intervention strategies aimed at addressing the identified needs and fostering an inclusive and supportive learning environment.

**Disclosure of Interest:**

None Declared

